# Predictive ability of CT findings in the differentiation of complicated and uncomplicated appendicitis: a retrospective investigation of 201 patients undergone appendectomy at initial admission

**DOI:** 10.1186/s13244-021-01086-3

**Published:** 2021-10-21

**Authors:** Jidapa Iamwat, Wanwarang Teerasamit, Piyaporn Apisarnthanarak, Napakadol Noppakunsomboon, Rathachai Kaewlai

**Affiliations:** 1 Division of Diagnostic Radiology, Department of Radiology, Faculty of Medicine Siriraj Hospital, Mahidol University, 2 Wanglang Rd, Bangkok Noi, Bangkok, 10700 Thailand; 2 Division of Acute Care Surgery, Department of Surgery, Faculty of Medicine Siriraj Hospital, Mahidol University, 2 Wanglang Rd, Bangkok Noi, Bangkok, 10700 Thailand

**Keywords:** Appendicitis, Adult, Retrospective studies, Computed tomography, Diagnostic performance

## Abstract

**Background:**

Paradigm shift toward nonoperative management (NOM) of adult appendicitis has made computed tomography (CT) more important than ever, particularly in differentiating complicated from uncomplicated disease. Complete surgical and pathological data of appendicitis in a place where appendectomy at initial admission is a standard of care would allow retrospective review of preoperative CT for performance and predictive ability in identifying those that may benefit from NOM in the future.

**Results:**

The study included 201 CT scans of consecutive adult patients who presented for appendectomy at initial admission with pathologically confirmed acute appendicitis. Complicated appendicitis referred to gangrene or perforation on pathological or operative findings. The overall CT sensitivity, specificity and accuracy for differentiation of complicated from uncomplicated appendicitis were 87.2%, 75.7% and 81.1%, respectively. The most sensitive CT findings of complicated appendicitis were mucosal enhancement defect (83.2%; 95% CI 74.1–90.0) and moderate-to-severe periappendiceal fat stranding (96.8%; 95% CI 91.1–99.3), both independently predictive of complicated appendicitis with adjusted odds ratios (ORs) of 4.62 (95% CI 1.86–11.51) and 4.41 (95% CI 1.06–18.29), respectively. Phlegmon, fluid collection, extraluminal appendicolith, periappendiceal air and small bowel dilatation had specificity of 98.1–100%. Intraluminal appendicoliths were found more frequently in complicated appendicitis (52.6% vs. 22.6%) but not predictive for this diagnosis. Independent clinical predictors of complicated appendicitis were lack of pain migration (OR 2.06), neutrophilia ≥ 82% (OR (2.87) and symptoms ≥ 24 h (OR 5.84).

**Conclusions:**

CT findings were highly accurate in differentiating complicated from uncomplicated appendicitis among patients undergone appendectomy at initial admission.

## Key points

- CT features allow accurate differentiation between complicated and uncomplicated appendicitis.
- Mucosal enhancement defect and moderate-to-severe periappendiceal fat stranding independently predict complicated appendicitis.
- Correct differentiation of complicated-vs-uncomplicated appendicitis benefits selection process for nonoperative management.

## Background

Acute appendicitis is the most common cause of surgical abdomen with an incidence of 90–100 per 100,000 population [1] or a lifetime prevalence of approximately 7% [2]. Cross-sectional imaging is a very useful noninvasive method for the evaluation of patients suspected of having acute appendicitis as history and physical examination may not be specific. Many other possible causes of pain in the right iliac fossa that can be diagnosed with ultrasound (US) or computed tomography (CT) are numerous, many of which are nonsurgical entities such as Crohn's disease, infectious enterocolitis, typhlitis, epiploic appendagitis, omental infarction, mesenteric adenitis and pelvic inflammatory disease [3, 4]. Therefore, a definitive diagnosis—usually derived at imaging—becomes essential to establish the need for surgery.

Strategies for imaging patients with suspected appendicitis usually revolve around clinical probability of the disease (using one of many available clinical prediction/decision rules), in which—if imaging is to be performed—this may start with CT first, or US first with conditional CT when US is inconclusive [5]. Specific patients’ demographics put value of an US-first strategy in children and women of child-bearing age as differential diagnoses are often vast and also to reduce radiation burden [2, 4, 5]. For the rest of population, CT is often considered the most appropriate first imaging test owing to its high accuracy for both diagnosis, characterization of appendicitis and strong ability to suggest alternative diagnosis [4], but value of the US-first strategy with conditional CT or even US re-evaluation after an equivocal CT cannot be understated [5, 6].

Once the diagnosis of acute appendicitis is made, decision to operate relies on whether the disease is locally complicated with phlegmon and abscess. Those with this complication typically require intravenous antibiotics with or without drainage, followed by interval appendectomy. The rest (i.e., uncomplicated, complicated disease with gangrene and perforation) classically receives urgent appendectomy during the same admission [7]. Although this approach has long been the mainstay treatment of acute appendicitis, there is a paradigm shift toward nonoperative management (NOM) given new evidence showing a high success rate, comparable 30-day health status and patient acceptance of antibiotic-first approach for uncomplicated appendicitis [8–11]. However, complication-free treatment success rate of this approach (68.4%) is still inferior to that of surgery (89.8%) with a failed rate of NOM during primary hospitalization in approximately 8% of cases [12]. Therefore, the World Society of Emergency Surgery (WSES) Jerusalem guidelines currently recommend NOM as a safe alternative to surgery only in selected patients. Importantly, the WSES guidelines specifically point out the issue of patient selection and exclusion of those with complicated appendicitis (i.e., gangrenous or perforated disease) as a factor limiting the success of NOM [13].

Although clinical appearance and scoring systems such as Alvarado score are generally sufficient to exclude acute appendicitis, they have limited usefulness in discrimination between uncomplicated and complicated appendicitis [19–21]. For this reason, this task heavily relies on contrast-enhanced CT in which a diagnosis of uncomplicated appendicitis can be made when there is no evidence of gangrene, perforation, periappendiceal abscess, appendicolith, or suspected tumor [10, 16].

A meta-analysis published in 2018 includes 23 articles deemed of an acceptable quality in evaluating CT performance of individual findings in distinguishing uncomplicated and complicated appendicitis. Authors found that most CT findings of complicated appendicitis are relatively highly specific (> 70% specificity) but not sensitive (14–59%) with only one finding being highly sensitive (94%) but nonspecific (40%) [17]. This further affirms a wide overall sensitivity of CT between 64 and 88% reported previously [18–24]. A recently published article reveals an astonishingly high level of overlooked appendiceal perforation at CT when using pathology as a reference standard [25], raising a further question about CT accuracy for distinction of uncomplicated and complicated appendicitis.

Although our practice still accepts appendectomy as a standard of care in acute appendicitis without clinical and/or CT signs of contained complication (i.e., abscesses, phlegmons), data specific to this patient group will help filling a knowledge gap by identifying diagnostic performance of both clinical features and CT findings among those typically opted for urgent appendectomy. Results will help improve a process of patient selection for NOM by allowing more accurate differentiation between uncomplicated and complicated appendicitis. Our aim was to explore clinical and CT findings in detail and identify a finding or combination of findings to help differentiating these two conditions among those deemed for surgery at their initial admission.

## Material and methods

### Study design and patients

**Fig. 1:**
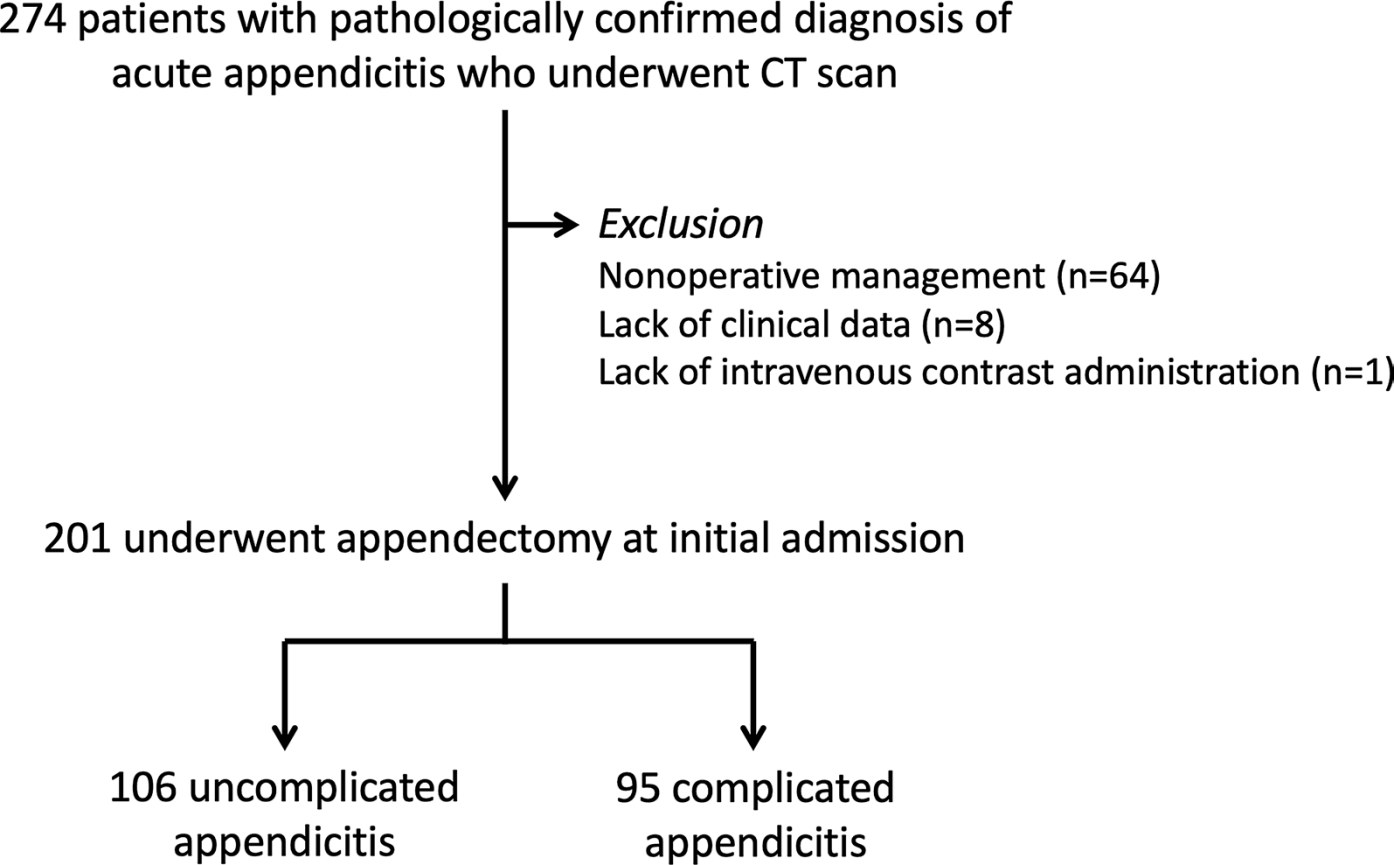
Flowchart of selection of study patients

This retrospective single-center study was approved by our Institutional Review Board (protocol No. 519/2563(IRB3) with COA No. Si 813/2020). The requirement for informed consent was waived because of the retrospective nature of this study. Between October 2016 and December 2019, 274 adult patients (age ≥ 18 years) with a final diagnosis of acute appendicitis underwent CT of the abdomen and pelvis at our urban academic hospital. Those who had CT without intravenous contrast administration (*n* = 1), were pregnant (*n* = 0) or lacked clinical data (*n* = 8) were excluded from the investigation. We excluded patients with nonsurgical management at initial admission (all cases were diagnosed as appendiceal abscess; *n* = 64). The final study population comprised of 201 patients (Fig. 1), which met the sample size calculated initially based on prevalence of complicated appendicitis of at least 25% with 95% confidence interval and 6% allowable error.

### Image acquisition

All CT scans were acquired on a 64-slice MDCT (LightSpeed VCT, GE Healthcare and Discovery CT750 HD, GE Healthcare) or a 256-slice MDCT (Revolution CT, GE Healthcare) with intravenous administration of 100 mL of nonionic contrast media at 2 mL/s via injectors. Scan parameters were set to 120 kVp and 300 mAs for 64-MDCT or 250 mAs for 256-MDCT, respectively. Oral and rectal contrast media were not administered. Each scan was obtained in an unenhanced phase, followed by a portovenous phase (approximately 80 s after contrast administration) with an axial slice thickness of 1.25 mm, covering from hepatic dome to pubic symphysis. All images were transferred to a Picture Archiving and Communication System (PACS; Synapse, Fujifilm Corporation) for viewing.

### Original reports

Original radiology reports were interpreted by a group of radiologists (*n* = 15) with an experience between 1 and 24 years. One hundred and thirty-five reports were finalized by abdominal radiologists, 60 by body imaging fellows and 6 by body imaging radiologists. The original reports were categorized into two groups, uncomplicated and complicated acute appendicitis. The former represented those reported as acute appendicitis without complication or early acute appendicitis. The latter included specific terms of gangrenous, focal wall defect, focal wall disruption, phlegmon, perforation, fluid collection, or acute appendicitis with complication.

### Image re-interpretation and definitions of CT findings

Two radiologists (one in abdominal and another in emergency subspecialty)—both with 20 years of experience—independently re-reviewed CT scans of all patients on a PACS workstation with ability to adjust window level/width and image orientation. CT findings were categorized into 3 groups: appendiceal, periappendiceal and intestinal findings. The appendiceal findings include mucosal hyperenhancement and defect, intraluminal content and appendicolith. Periappendiceal findings were surrounding fat stranding, phlegmon, fluid collection, extraluminal appendicolith, periappendiceal air, periappendiceal fluid and ascites. Intestinal findings consisted of small bowel dilatation and small bowel thickening. Reviewers also made a final impression whether they thought the overall findings were consistent with uncomplicated or complicated appendicitis.

The definitions of each CT finding are provided in Table 1 [17, 26, 27]. The radiologists were blinded to clinical data and pathological results. All disagreements between two radiologists were resolved by a third radiologist—subspecialized in abdominal imaging—with 24 years of experience.

**Table 1:** Definitions of CT findings

CT findings	Definitions
Mucosal hyperenhancement	Mucosal enhancement of appendix was compared to that of normal small bowel loops
Mucosal enhancement defect	Focal absence of mucosal enhancement of appendix
Fluid collection	Extraluminal fluid collection with/without contrast-enhanced wall
Periappendiceal fluid	Extraluminal fluid around the appendix without encapsulation
Periappendiceal fat stranding	Increased attenuation of fat surrounding appendix. Just perceptible (thickness, 1–2 mm), mild; others, moderate–severe
Phlegmon	A mass-like soft tissue density within the right lower abdominal quadrant surrounding inflamed appendix
Ascites	Free fluid with is considered larger than a physiologic amount
Small bowel dilatation	Small bowel caliber of larger than 2.5 cm
Appendicolith	A calcific focus inside appendiceal lumen measuring ≥ 2 mm in diameter, or outside the lumen within fluid or fluid collection
Tip appendiceal diameter	Diameter of appendix measured at within 1 cm of the tip

## Reference standards

The diagnosis of acute appendicitis was made based on histopathological results. Complicated appendicitis included those with gangrene or perforation [1]. The diagnosis of gangrene was made with histopathology, while the diagnosis of perforation was documented either on histopathology or surgical operative findings.

### Statistical analysis

Categorical variables such as gender, symptoms, signs, and CT findings were presented as number or percentage. Continuous data such as age, body mass index (BMI), temperature, duration from CT to surgery, duration of symptoms, and duration to antibiotics were reported as mean (standard deviation) or median (range) depending on data distribution.

Clinical and CT findings of the two groups were compared using Chi-square test (for categorical variables) and *t*-test or Mann–Whitney *U* test (for continuous variables). Univariate and multivariate analyses were performed. Logistic regression was used to determine the odds ratio for independent predictors. Statistical Package for Social Sciences (SPSS, version 23, IBM) was utilized for these analyses. The threshold for assessing statistical significance was set to 0.05. Interobserver agreement between two radiologists was calculated and found to be 0.67 (kappa; range, 0.57–0.77). CT performance was derived from a 2 × 2 table and reported as sensitivity, specificity, accuracy, positive predictive value (PPV), negative predictive value (NPV), positive likelihood ratio (PLR) and negative likelihood ratio (NLR).

## Results

### Patients

The study group comprised of 201 patients, in whom 95 had complicated appendicitis (18 gangrenous appendicitis and 77 perforated appendicitis). There was no statistically significant difference in patient characteristics between the uncomplicated and complicated groups in terms of gender, BMI, Alvarado score, duration to the first dose of antibiotics, and duration from CT scan to surgery. The average age, temperature, percentage of neutrophil count and duration of symptoms of those with complicated appendicitis were significantly higher than those with uncomplicated appendicitis (Table 2).

**Table 2:** Patient characteristics

Characteristics	All patients (*n* = 201)	Uncomplicated appendicitis (*n* = 106)	Complicated appendicitis (*n* = 95)	*p* value
Gender				
Male	68 (33.8)	34 (32.1)	34 (35.8)	0.578
Female	133 (66.2)	72 (67.9)	61 (64.2)	
Age (years), mean ± SD	53.6 ± 19.9	49.3 ± 19.4	58.5 ± 19.6	0.001
BMI, mean ± SD	23.5 ± 4.86	23.7 ± 4.4	23.3 ± 3.4	0.556
< 18.5	19 (9.5)	11 (10.4)	8 (8.4)	0.412
18.5–24.9	119 (59.2)	57 (53.8)	62 (65.3)	
> 25	63 (31.3)	38 (35.8)	25 (26.3)	
Symptoms and signs				
RLQ tenderness	196 (97.5)	104 (98.1)	92 (96.8)	0.669
Temperature (°C)	37.4 ± 0.8	37.3 ± 0.7	37.6 ± 0.9	0.002
Rebound tenderness	92 (45.8)	44 (41.5)	48 (50.5)	0.200
Migration of pain	85 (42.3)	53 (50.0)	32 (33.7)	0.019
Anorexia	99 (49.3)	45 (42.5)	54 (56.8)	0.042
Nausea or vomiting	117 (58.2)	57 (53.8)	60 (63.2)	0.178
Laboratory results				
WBC count (cells/mm^3^)	13,549 (3870–26,500)	12,810 (4680–24,670)	13,340 (3870–26,560)	0.470
Neutrophilia (%)	80.7 (28.6–95.7)	80.7 (38.0–95.7)	84.8 (28.6–95.2)	0.003
Alvarado score				
0–4	17 (8.5)	9 (8.5)	8 (8.4)	0.071
5–8	162 (80.6)	91 (85.8)	71 (74.7)	
9–10	22 (10.9)	6 (5.7)	16 (16.8)	
Duration of symptom (h), median (min, max)	24 (2–480)	16 (2–72)	48 (4–480)	< 0.001
Time from CT to surgery (h), median (min, max)	3 h 48 m (20 m–30 h)	3 h 48 m (24 m–30 h)	3 h 24 m (20 m–30 h)	0.651
< 6 h	157 (78.1)	79 (74.5)	78 (82.1)	0.574

RLQ: Right lower quadrant

Those in brackets represent percentage unless specified otherwise

### CT findings

The details of CT findings are demonstrated in Table 3 and illustrated in Figs. 2, 3, 4, 5, 6, 7, 8 and 9.

**Table 3:** CT characteristics

CT findings	All patients (*n* = 201)	Uncomplicated appendicitis (*n* = 106)	Complicated appendicitis (*n* = 95)	*p* value
Appendiceal diameter (mm)	12.0 ± 2.7	11.1 ± 2.3	13.1 ± 2.8	< 0.001
Tip appendiceal diameter (*n* = 186) (mm)	10.5 ± 2.7	9.8 ± 2.3	11.4 ± 3.0	< 0.001
Mucosal hyperenhancement	91 (45.3)	45 (42.5)	46 (48.4)	0.396
Mucosal enhancement defect	101 (50.2)	22 (20.8)	79 (83.2)	< 0.001
Intraluminal appendicolith	74 (36.8)	24 (22.6)	50 (52.6)	< 0.001
Appendicolith causing obstruction	57 (28.6)	18 (17.1)	39 (41.5)	< 0.001
Fat stranding				< 0.001
None	5 (2.5)	4 (3.8)	1 (1.1)	
Mild	43 (21.4)	41 (38.7)	2 (2.1)	
Moderate–severe	153 (76.1)	61 (57.5)	92 (96.8)	
Phlegmon	11 (5.5)	1 (0.9)	10 (10.5)	0.003
Fluid collection	24 (11.9)	1 (0.9)	23 (24.2)	< 0.001
Size (mm)	34.7 ± 14.9	21.4 ± 3.7	35.8 ± 14.9	0.193
Extraluminal appendicolith	6 (3.0)	0 (0.0)	6 (6.3)	0.010
Periappendiceal air	41 (20.4)	0 (0.0)	41 (43.2)	< 0.001
Periappendiceal fluid	103 (51.2)	30 (28.3)	73 (76.8)	< 0.001
Ascites	60 (29.9)	16 (15.1)	44 (46.3)	< 0.001
Small bowel dilatation	17 (8.5)	2 (1.9)	15 (15.8)	< 0.001
Diameter (cm)	3.5 ± 0.4	3.4 ± 0.4	3.5 ± 0.5	0.508
Small bowel thickening	60 (29.9)	13 (12.3)	47 (49.5)	< 0.001

Those in brackets represent percentage unless specified otherwise

**Fig. 2:**
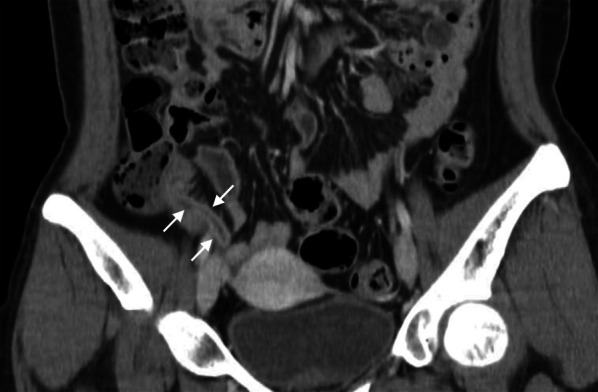
Uncomplicated appendicitis. A coronal-reformatted CT image of a 35-year-old woman presenting with a 7-h onset of right lower quadrant pain, elevated white blood cell counts (11,590 cells/mm^3^) and neutrophilia (80.3% neutrophils) reveals a dilated appendix (*arrows*) with mucosal hyperenhancement and fluid-filled appendiceal lumen. Suppurative appendicitis was confirmed at surgery and histopathology

**Fig. 3:**
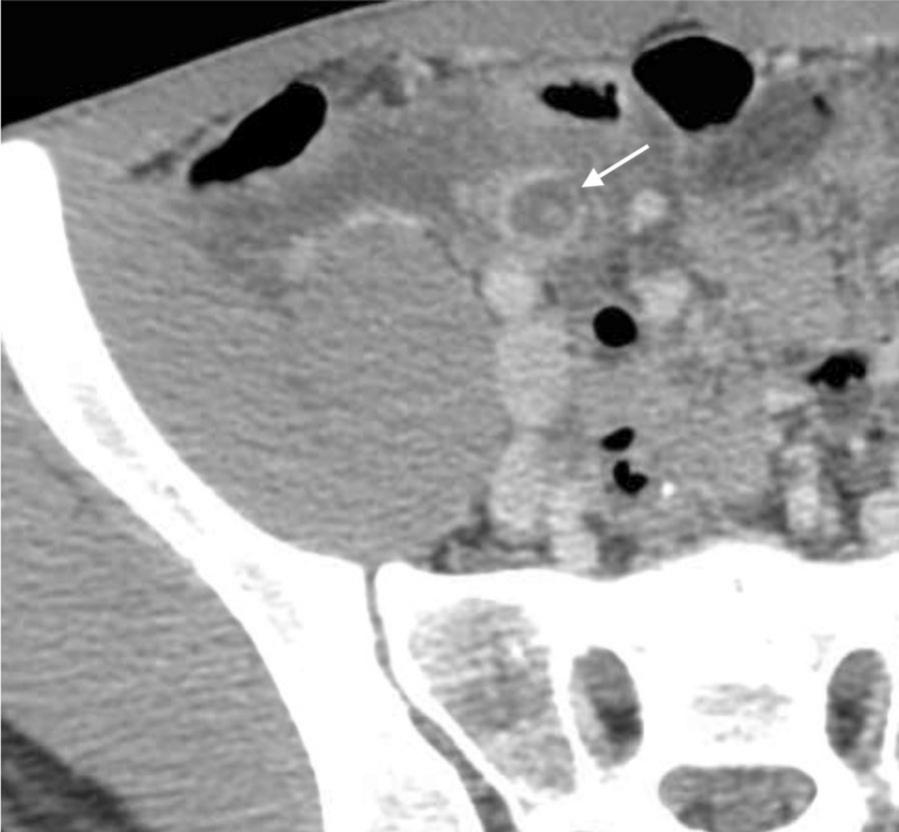
Mucosal enhancement defect of appendix. An axial CT image of a 20-year-old man presenting with a 9-h onset of right lower quadrant pain, elevated white blood cell counts (19,150 cells/mm3) and neutrophilia (85% neutrophils) shows a dilated appendix with focal defect at the anteromedial wall (*arrow*). Gangrenous appendicitis was confirmed at surgery and histopathology

**Fig. 4:**
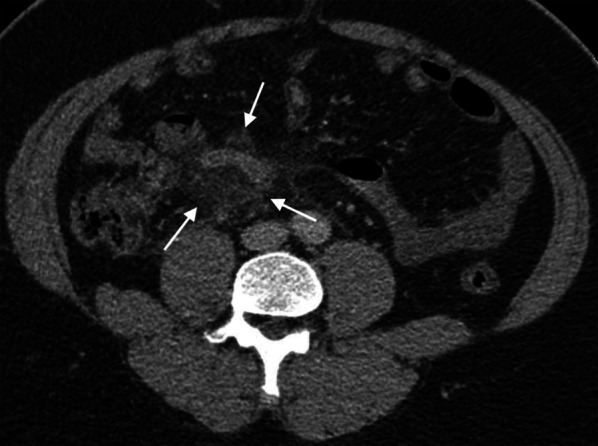
Moderate-to-severe periappendiceal fat stranding. An axial CT image of a 40-year-old man presenting with a 48-h onset of right lower quadrant pain, elevated white blood cell counts (14,010 cells/mm^3^) and neutrophilia (85.2% neutrophils) shows a dilated appendix with mucosal hyperenhancement and moderate-to-severe periappendiceal fat stranding (*arrows*). Gangrenous appendicitis was confirmed at surgery and histopathology

**Fig. 5:**
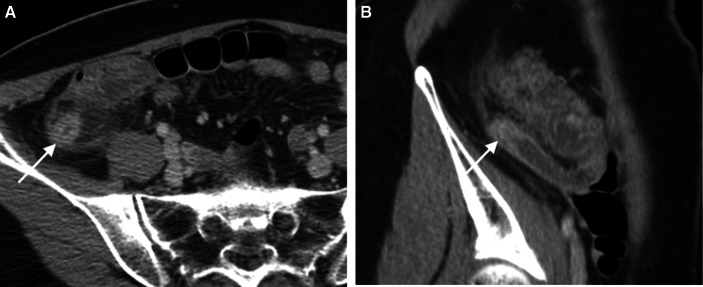
Mucosal hyperenhancement. Axial (**a**) and sagittal-reformatted (**b**) CT images of a 58-year-old woman presenting with right lower quadrant pain for 16 h, elevated white blood cell counts (10,060 cells/mm^3^) and neutrophilia (71.3% neutrophils) show an enlarged appendix with wall thickening and hyperenhancement at its distal end (arrow). Note moderate-to-severe periappendiceal fat stranding. Gangrenous appendicitis was confirmed at surgery and histopathology

**Fig. 6:**
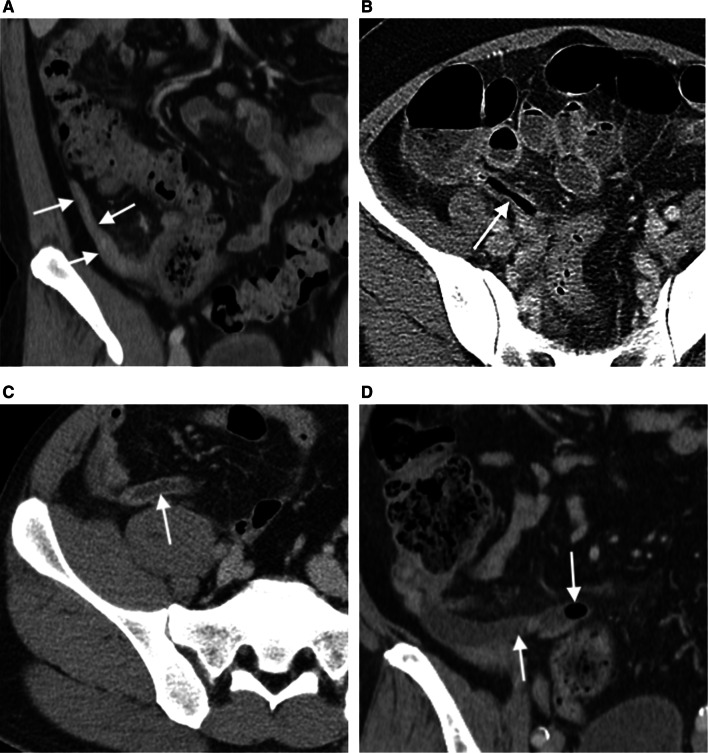
Four patterns of intraluminal content in appendicitis. Axial and coronal-reformatted CT images of four different patients reveal appendices completely obliterated (**a**), filled entirely with air (**b**), fluid (**c**) or air mixed with fluid and enteric content (**d**). These four patients were diagnosed with uncomplicated (the first two), perforated and gangrenous at surgery and histopathology, respectively

**Fig. 7:**
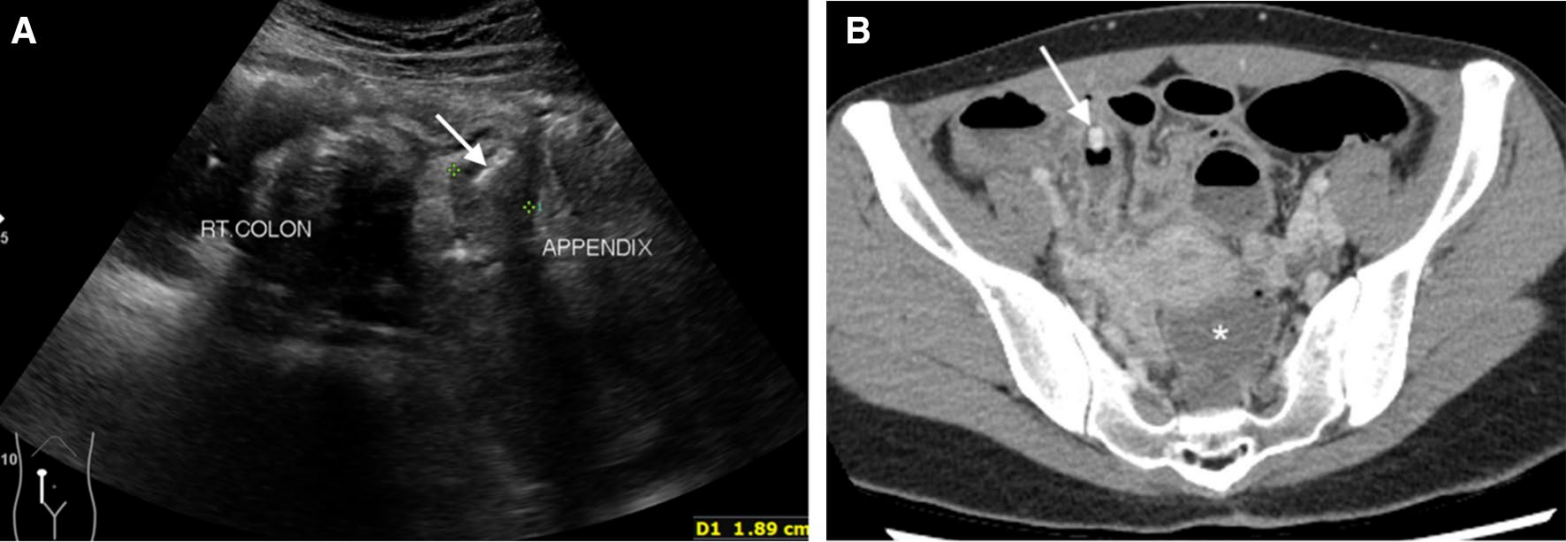
Intraluminal appendicolith. An ultrasound image **a** of a 35-year-old woman presenting with right lower abdominal pain for 1 day, elevated white blood cell counts (23,790 cells/mm^3^) and neutrophilia (93.3% neutrophils) shows a dilated appendix (arrows) with an intraluminal hyperechoic focus representing appendicolith. An obstructive appendicolith with acute appendicitis is confirmed on subsequent CT **b** that also reveals fluid in the cul-de-sac (asterisk) and peritoneal enhancement. Perforated appendicitis with turbid intraperitoneal fluid was confirmed at surgery

**Fig. 8:**
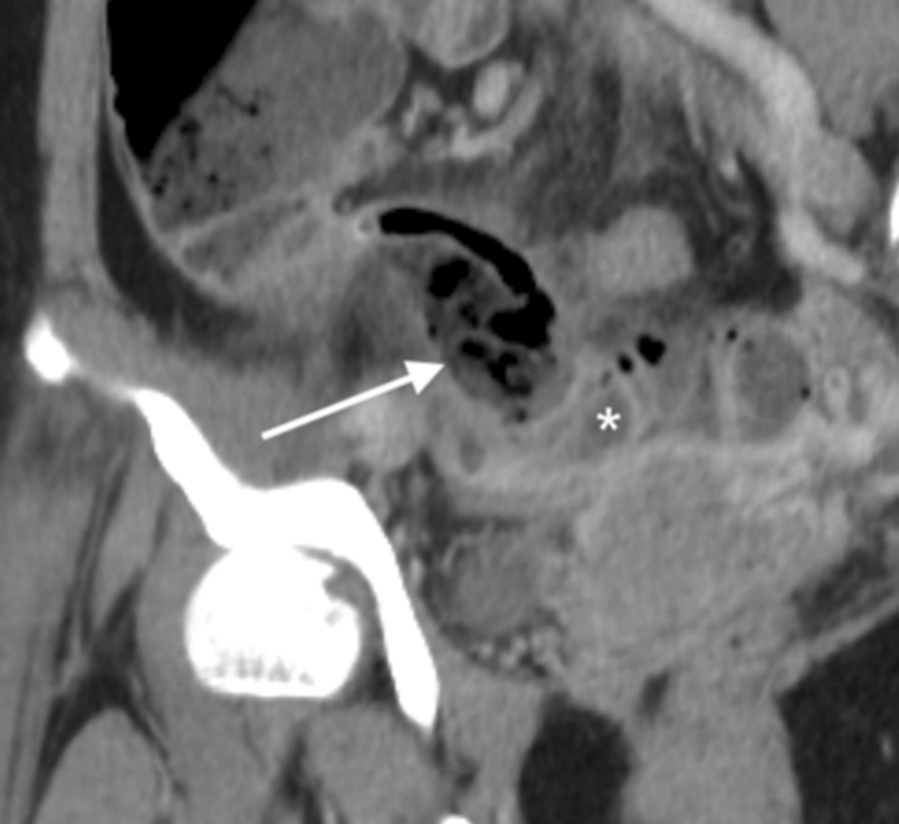
Fluid and air collection. A coronal-reformatted CT image of a 54-year-old woman presenting with right lower abdominal pain and fever for 20 h, elevated white blood cell counts (18,060 cells/mm^3^) and neutrophilia (92.2% neutrophils) shows an extraluminal air bubbles mixed with fluid and enteric content (arrows) inferior to an inflamed appendix. Note moderate-to-severe periappendiceal fat stranding with nearby fluid-filled nondilated small bowel loops. Perforated appendicitis was confirmed at surgery and histopathology

**Fig. 9:**
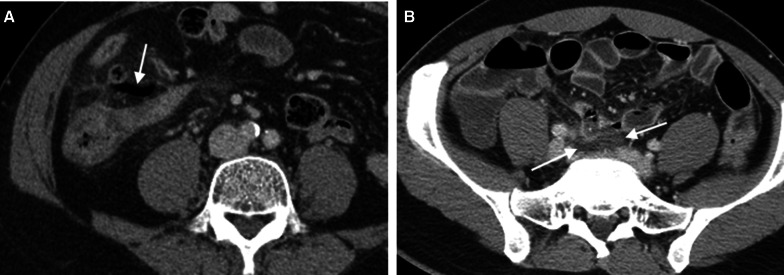
Periappendiceal air and fluid. Axial CT images of two different patients show extraluminal air within mesoappendix (arrow in **a**) and extraluminal periappendiceal fluid (arrow in **b**). The former patient is a 73-year-old man who presents with abdominal pain and fever, slightly elevated white blood cell counts (11,370 cells/mm^3^) and neutrophilia (88.0% neutrophils). The latter is a 39-year-old-man presenting with right lower abdominal pain for 1 day, elevated white blood cell counts (17,100 cells/mm^3^) and neutrophilia (86.8% neutrophils). Both have a confirmed diagnosis of perforated appendicitis

### Primary analysis

For differentiation between complicated and uncomplicated appendicitis, CT had sensitivity of 87.2% (95%CI; 78.7–93.2), specificity of 75.7% (95%CI; 66.4–83.4), and accuracy of 81.1% (95%CI; 74.9–86.2). Based on the original radiology reports, sensitivity, specificity and accuracy were 74.5% (95%CI; 64.4–82.9), 86% (95%CI; 77.9–91.9) and 80.6% (95%CI; 74.4–85.8), respectively.

The sensitivities, specificities and other diagnostic values of each CT finding are demonstrated in Table 4. Mucosal enhancement defect had the highest accuracy (80.6%) with moderate sensitivity (82.9%), specificity (78.5%) and NPV (84%). The most sensitive finding was moderate-to-severe fat stranding (96.8%) with a highest NPV (93.8%), but it had poor specificity (42.5%). The high specificities between 99.1 and 100% were detected in extraluminal appendicolith, phlegmon, small bowel dilatation, fluid collection, and periappendiceal air, but they had low sensitivity of 6.3%, 10.5%, 15.8%, 24.2% and 43.2%, respectively.

**Table 4:** Performance of each CT finding in differentiating complicated from uncomplicated appendicitis

CT findings	Sensitivity	Specificity	Accuracy	PLR	NLR	PPV	NPV	*p* value
Appendiceal diameter ≥ 12 mm^a^	61.7	65.1	63.5	1.8	0.6	61.1	65.7	< 0.001
	(51.1–71.5)	(55.2–74.1)	(56.4–70.2)	(1.3–2.4)	(0.4–0.8)	(53.6–68.0)	(58.9–72.0)	
Tip appendiceal diameter ≥ 10 mm^b^ (*n* = 186)	68.3	54.8	60.8	1.5	0.6	54.4	68.7	< 0.001
	(57.1–78.1)	(44.7–64.7)	(53.3–67.8)	(1.2–2.0)	(0.4–0.8)	(47.9–60.7)	(60.4–75.9)	
Mucosal hyperenhancement	10.9	57.6	35.5	0.3	1.6	18.7	41.9	0.396
	(4.1–22.3)	(47.6–67.1)	(28.1–43.4)	(0.1–0.6)	(1.3–1.9)	(9.5–33.6)	(37.4–46.5)	
Mucosal enhancement defect	83.2	79.3	81.1	4.0	0.2	78.2	84.0	< 0.001
	(74.1–90.0)	(70.3–86.5)	(75.0–86.3)	(2.7–5.9)	(0.1–0.3)	(71.0–84.0)	(76.9–89.2)	
Intraluminal appendicolith	52.6	77.4	65.7	2.3	0.6	67.5	64.6	< 0.001
	(42.1–63.0)	(68.2–84.9)	(58.7–72.2)	(1.6–3.5)	(0.5–0.8)	(58.3–75.7)	(59.0–69.8)	
Appendicolith causing obstruction	41.5	82.9	63.3	2.4	0.7	68.4	61.3	< 0.001
	(31.4–52.1)	(74.3–89.5)	(56.2–70.0)	(1.5–3.9)	(0.6–0.9)	(57.2–77.9)	(56.6–65.7)	
Fat stranding; moderate–severe	96.8	42.5	68.2	1.7	0.1	60.1	93.8	< 0.001
	(91.1–99.3)	(32.9–52.4)	(61.2–74.5)	(1.4–2.0)	(0.0–0.2)	(56.1–64.1)	(82.8–97.9)	
Phlegmon	10.5	99.1	57.2	11.2	0.9	90.9	55.3	0.003
	(5.2–18.5)	(94.9–100.0)	(50.1–64.2)	(1.5–85.6)	(0.8–1.0)	(56.6–98.7)	(53.5–57.0)	
Fluid collection	24.2	99.1	63.7	25.7	0.8	95.8	59.3	< 0.001
	(16.0–34.1)	(94.9–100.0)	(56.6–70.3)	(3.5–186.4)	(0.7–0.9)	(76.0–99.4)	(56.5–62.1)	
Extraluminal appendicolith	6.3	100.0	55.7	N/A	0.9	100.0	54.4	0.010
	(2.4–13.2)	(96.6–100.0)	(48.6–62.7)		(0.9–1.0)		(53.1–55.7)	
Periappendiceal air	43.2	100.0	73.1	N/A	0.6	100.0	66.3	< 0.001
	(33.0–53.7)	(96.6–100.0)	(66.5–79.1)		(0.5–0.7)		(62.2–70.0)	
Periappendiceal fluid	76.8	71.7	74.1	2.7	0.3	70.9	77.6	< 0.001
	(67.1–84.9)	(62.1–80.0)	(67.5–80.0)	(2.0–3.8)	(0.2–0.5)	(63.8–77.1)	(70.2–83.6)	
Ascites	46.3	84.9	66.7	3.1	0.6	73.3	63.8	< 0.001
	(36.0–56.9)	(76.7–91.1)	(59.7–73.1)	(1.9–5.1)	(0.5–0.8)	(62.5–81.9)	(59.0–68.4)	
Small bowel dilatation	15.8	98.1	59.2	8.4	0.9	88.2	56.5	< 0.001
	(9.1–24.7)	(93.4–99.8)	(52.1–66.1)	(2.0–35.7)	(0.8–0.9)	(63.8–97.0)	(54.3–58.8)	
Small bowel thickening	49.5	87.7	69.7	4.0	0.6	78.3	66.0	< 0.001
	(39.1–59.9)	(79.9–93.3)	(62.8–75.9)	(2.3–7.0)	(0.5–0.7)	(67.6–86.2)	(61.1–70.5)	

^a^Area under ROC curve 0.687 (0.613–0.760)

^b^Area under ROC curve 0.658 (0.580–0.736)

PLR: Positive likelihood ratio, NLR: negative likelihood ratio, PPV: positive predictive value, NPV: negative predictive value, N/A: not available

Those in brackets represent 95% confidence interval

### Prediction of complicated appendicitis

The univariate and multivariate logistic regression analyses were conducted in two sessions. First, demographic data were tested. In univariate analysis, statistically significant factors in discriminating uncomplicated and complicated appendicitis were age ≥ 50 years, temperature ≥ 37 °C, migration of pain, neutrophilia ≥ 82% and duration of symptom ≥ 24 h. An adjusted odds ratio from multivariate logistic regression analysis showed three statistically significant factors, which were lack of pain migration (adjusted OR of 2.06 with 95%CI of 1.03–4.13; *p* = 0.04), neutrophilia ≥ 82% (adjusted OR of 2.87 with 95% CI of 1.42–5.81; *p* 0.003) and duration of symptoms ≥ 24 h (adjusted OR of 5.84 with 95% CI of 2.86–11.96; *p* < 0.001). Details are provided in Table 5.

**Table 5:** Multivariate regression analyses of demographic^1^ and CT^2^ data

Characteristics	Adjusted OR (95%CI)	*P* value
Age ≥ 50 years	1.67 (0.86–3.24)	0.132
Symptoms and signs		
Temperature ≥ 37 °C	1.85 (0.90–3.77)	0.093
Lack of pain migration	2.06 (1.03–4.13)	0.040
Anorexia	1.25 (0.61–2.57)	0.539
Laboratory results		
Neutrophilia ≥ 82%	2.87 (1.42–5.81)	0.003
Alvarado score		
0–4	1	
5–8	0.83 (0.25–2.77)	0.757
9–10	1.90 (0.33–10.95)	0.473
Duration of symptom ≥ 24 h	5.84 (2.86–11.96)	< 0.001
CT findings		
Appendiceal diameter > 12 mm	1.47 (0.58–3.72)	0.417
Tip appendiceal diameter > 10 mm	1.18 (0.46–3.00)	0.728
Mucosal enhancement defect	4.62 (1.86–11.51)	0.001
Intraluminal appendicolith	1.64 (0.39–6.85)	0.499
Appendicolith causing obstruction	0.78 (0.16–3.71)	0.751
Fat stranding; moderate–severe	4.41 (1.06–18.29)	0.041
Fluid collection	4.45 (0.49–40.74)	0.187
Periappendiceal fluid	1.22 (0.47–3.20)	0.687
Ascites	1.98 (0.73–5.40)	0.182
Small bowel dilatation	1.31 (0.19–8.93)	0.786
Small bowel thickening	1.20 (0.45–3.16)	0.720

^1^Nagelkerke *R*^2^ = 0.336; overall accuracy 73.1%

^2^Nagelkerke *R*^2^ = 0.563; overall accuracy = 79.5%

Second, CT findings were tested. In univariate analysis, majority of findings showed statistical significance except mucosal hyperenhancement. Phlegmon, extraluminal appendicolith and periappendiceal air were not used in multivariate logistic regression analysis because of their low prevalence in the uncomplicated group. Two independent CT predictors were mucosal enhancement defect and moderate-to-severe fat stranding, which had adjusted ORs of 4.62 (95% CI of 1.86–11.51) and 4.41 (95% CI of 1.06–18.29), respectively (Table 5).

## Discussion

### Overall CT performance

The overall CT sensitivity in differentiating between complicated and uncomplicated appendicitis is 87.2%, which is comparable and on the upper end of that of prior investigations demonstrating 64–88% sensitivity. However, specificity of 75.7% is lower than those reported previously (85–99%) [18–23]. This may be explained by inclusion of both gangrenous and perforated appendicitis, and exclusion of those receiving NOM at initial admission (all having abscesses) in our study group. The latter would have been obvious at CT, while the former would be more difficult to diagnose or excluded based on CT findings as demonstrated in the study by Hong et al. [28]. In their investigation, upon a re-review of CT of patients designated as having “uncomplicated” appendicitis and treated with antibiotic (then failed), they found that about one-third actually had qualitative and quantitative hypoenhancement of appendiceal wall (i.e., findings of gangrenous appendicitis). In fact, even in comparison with perforated appendicitis only, previously believed excellent CT performance becomes highly questionable in the study by Gaskill et al. [25]. In this study, a re-review of 89 CT scans (48% with pathologically confirmed perforated appendicitis) by 15 abdominal imaging fellowship-trained radiologists found that 93% of perforations were overlooked. Of note, the operative notes were concordant with pathological reports in only 28% of cases. This raises a possibility that pathologically diagnosed perforations were minute, which may not be obvious at CT. Nevertheless, further exploration is highly necessary if we want to improve risk prediction for failure of treatment with antibiotic therapy in acute appendicitis. Dual-energy CT with low keV and iodine overlay images have been proven useful in this regard, providing a very high accuracy for diagnosing gangrenous appendicitis [29]. This might open a new frontier in CT imaging for detailed and accurate assessment of appendicitis.

We also tested ten CT findings used by Kim et al. [24] to suggest a diagnosis of complicated appendicitis based on presence of at least 1 out of 10 of these findings: contrast enhancement defect of the appendiceal wall, fluid collection, extraluminal air, intraluminal air, extraluminal appendicolith, intraluminal appendicolith, periappendiceal fat stranding (moderate-to-severe degree), periappendiceal fluid, ileus, and ascites. We found that their criteria had a very high sensitivity of 97.9%, comparable to their subjects (sensitivity 92% with 95% CI of 83–97%), making the criteria excellent as a screening method. However, their low specificity (in our investigation; 30.8% and theirs; 43% (95% CI: 31–55%)) would limit utilization of such criteria because many patients would be deterred from NOM.

### Performance of individual CT findings

Appendiceal mucosal enhancement defect has the highest sensitivity (82.9%), specificity (78.5%) and accuracy (80.6%) among any CT findings for differentiating complicated and uncomplicated appendicitis, with sensitivity much higher than those previously reported. A systematic review and meta-analysis of CT findings [17] reveal a pooled sensitivity of only 59% (95% CI: 40–75) and a pooled specificity of 96% (95% CI: 90–99). This may be explained by thin-sliced CT images in our investigation, which allow superior identification of findings such as mucosal enhancement defect than those of a lower image resolution [20, 21, 30, 31]. The previous studies using thicker slices [32, 33] show lower sensitivity for this task.

Highly specific signs for complication such as extraluminal appendicolith, phlegmon, small bowel dilatation, fluid collection, and periappendiceal air were observed in our investigation, in line with prior studies [21, 34, 35]. Interestingly, phlegmons and fluid collections were found in 5.5% and 11.9% of our patient population even though we excluded those deemed for initial nonoperative management. These patients underwent appendectomy at their initial admission, most likely based on clinical evaluation (i.e., nonlocalized peritonitis, progressive symptoms and signs). At pathology, almost all of them (33/35; 94.3%) had complicated appendicitis, affirming the strength of CT in diagnosing complications.

Negative predictive values were high to very high for two CT findings, which can be helpful to exclude complications. Based on our data, when there was only mild degree (or absence) of periappendiceal fat stranding, and smooth uninterrupted mucosal enhancement of the appendix, complicated appendicitis would be unlikely. This suggests that nonoperative management may be appropriate for such patients.

### Prediction of complicated appendicitis

Our findings of independent clinical predictors of complicated appendicitis being neutrophilia (≥ 82%) and ≥ 24-h duration of symptoms are consistent with those shown in studies by Eddama et al. [36] and Suh et al. [23], respectively. In terms of CT findings, independent predictors of complicated appendicitis in our investigation are mucosal enhancement defect and moderate-to-severe fat stranding, which are in line with the diagnostic model for differentiation between complicated and uncomplicated appendicitis proposed by Kim et al. [37]. These two CT findings are 83.2–96.8% sensitive and have odds ratios of 4.41–4.62 in identifying complicated appendicitis. Table 6 presents odds ratios of these five independent variables found to be statistically significant (*p* < 0.05) and predictive (values above and not overlapping the null value) of this condition based on our multivariate regression analysis. Based on these variables, if a patient with acute appendicitis has all factors combined, the odds of him/her having complicated appendicitis would be 19.59 times over those without these factors.

**Table 6:** Summary of statistically significant factors predictive of complicated appendicitis

Factors	Odds ratios^a^
Clinical characteristics	
Lack of pain migration	2.06
Neutrophilia ≥ 82%	2.87
Duration of symptom ≥ 24 h	5.84
CT findings	
Mucosal enhancement defect	4.62
Moderate-to-severe fat stranding	4.41

^a^Odds ratios of those with 95% CIs above and not overlapping the null value, and a *p* value of less than 0.05 according to the multivariate regression analysis

The presence of appendicolith is associated with complicated appendicitis in our univariate analysis but—in contrary to previous reports—it is not statistically significant in the multivariate regression analysis. Appendicoliths have been found significantly more frequent among those with acute appendicitis, associated with increased inflammation, risk of perforation, and considered one of the risk factors for complicated appendicitis [13, 38]. Their presence is among an exclusion from clinical trials of NOM in acute appendicitis such as the Appendicitis Acuta (APPAC) trials [9, 10, 39]. In our investigation, intraluminal appendicolith was found in 74 out of 201 patients with this sign alone showing sensitivity, PPV and PLR of 52.6%, 67.5% and 2.3, respectively, to differentiate complicated from uncomplicated appendicitis. When considered only obstructing appendicolith, the sensitivity drops to 41.5%, while the PPV and PLR increase only slightly to 68.4% and 2.4, respectively. Nevertheless, appendicoliths are still a likely risk factor for failed NOM. A recent randomized trial by the Comparison of Outcomes of antibiotic Drugs and Appendectomy (CODA) collaborative comparing antibiotic with appendectomy [8] that included patients with appendicolith appendicitis but no overt perforation in their study group has revealed a higher risk for appendectomy and for complication (site-related complication and drainage procedure) in this subgroup.

Our investigation is limited by a retrospective nature. The sample size is relatively small although it reaches the pre-calculated level. There was no standard algorithm for selection of patients with suspected appendicitis for imaging; however, the use of Alvarado score is prevalent, and CT is the most common first-line imaging in adults suspected of having acute appendicitis in our practice. Since operative management of acute appendicitis is still a standard of care at our hospital, we assumed that almost all acute appendicitis without phlegmon and abscess including those with nonlocalized perforation would have been operated at an initial presentation. This way our cohort includes those having pathologically confirmed appendicitis. Although we have definitions of each CT finding, many are still subjective, but we tried to minimize bias by having two experts re-reviewed images with a third expert resolving all disagreements. This—however—does not reflect real-world practice in which a radiologist often makes an individual judgment that can potentially be less uniform. Our data support this notion as performance of an original CT results was slightly inferior to the reviews by a group of experts.

In conclusion, three clinical features and two CT findings allow accurate differentiation of complicated from uncomplicated appendicitis. These include lack of pain migration, neutrophilia (≥ 82%), duration of symptom (≥ 24 h), mucosal enhancement defect and moderate-to-severe periappendiceal fat stranding. A combination of these factors further increases the chance of having complicated appendicitis. This information may be helpful in creation of a clinical decision tree or templates for structured reporting in radiology.

## Data Availability

Datasets generated and/or analyzed during the current study are not publicly available due to their nature as personal health information but may be available from the corresponding author on reasonable request.

## Abbreviations

BMI: Body mass index
CI: Confidence interval
CODA: Comparison of outcomes of antibiotic drugs and appendectomy
CT: Computed tomography
MDCT: Multidetector CT
NLR: Negative likelihood ratio
NOM: Nonoperative management
NPV: Negative predictive value
OR: Odds ratio
PACS: Picture archiving and communication system
PLR: Positive likelihood ratio
PPV: Positive predictive value
WSES: World Society of Emergency Surgery
